# Case Report: Successful Treatment of Sarcoptic Mange in European Camelids

**DOI:** 10.3389/fvets.2021.742543

**Published:** 2021-09-14

**Authors:** Georgiana Deak, Barbara Moroni, Ana Maria Boncea, Luisa Rambozzi, Luca Rossi, Andrei Daniel Mihalca

**Affiliations:** ^1^Department of Parasitology and Parasitic Diseases, University of Agricultural Sciences and Veterinary Medicine of Cluj-Napoca, Cluj-Napoca, Romania; ^2^Department of Veterinary Sciences, University of Turin, Largo Paolo Braccini, Italy; ^3^Falcon Vet Veterinary Private Clinic, Bucharest, Romania

**Keywords:** alpaca (*Vicugna pacos*), llama (*Lama glama*), sarcoptic mange, *Sarcoptes scabiei*, avermectines

## Abstract

**Introduction:** Members of the Camelidae family are very adaptable mammals, originating from South America. More recently, they have become popular in Europe as pets or wool production farm animals. Their dermatological problems refer mainly to mange, of which sarcoptic mange represent the most clinically relevant form. There is a wide range of molecules effective against sarcoptic mange, but the treatment is very challenging due to the lack of efficiency and absorption.

**Methods:** Two cases from two different animal populations were described. A female alpaca from Romania with dermatological problems on the ears and two adult llamas, from Italy, both with intense pruritus. Combined treatment with amitraz and subcutaneous eprinomectin was administered for the alpaca, and 2% moxidectin was given to the llamas.

**Results:** In both cases, the mites were morphologically identified as *Sarcoptes scabiei*. For the alpaca, negative scrapings were found only after 8 weeks of combined treatment. For the llamas, after four doses of subcutaneous moxidectin, no mites were detected in scrapings and lively hair regrowth of previously alopecic areas was visible.

**Conclusion:** This paper aimed to present two clinical scenarios of sarcoptic mange in camelids, successfully treated with a combination of ectocides (topical amitraz and subcutaneous eprinomectin) and 2% subcutaneous moxidectin, respectively.

## Introduction

Alpacas and llamas are members of the Camelidae family and are very adaptable mammals, originating from South America, where they are kept for meat, hides, and high-quality wool. In the last decades, they have become increasingly popular in Europe as pets or wool production farm animals (1, 2). Due to the increased interest in South American Camelids, veterinarians should be aware of their husbandry practices, including their specific diseases. The most common conditions are related to the digestive tract, metabolic disorders, skin lesions and eye problems (2–4). Several types of mites have been reported in alpacas; however, sarcoptic mange seems to be the most severe in regard with the clinical manifestations (2, 5, 6). Lesions due to scabiosis appear first in the head area, and they can extend to the legs or even the whole body, manifested in the form of alopecia and excoriations (2). The intense pruritus can lead even to self-trauma and sometimes secondary bacterial infections can occur (2). Even though there is a wide range of ectoparasitic molecules effective against sarcoptic mange, in camelids, the treatment is more challenging as pour-on solutions are not well absorbed (7) and the systematic formulations are poorly efficient. Up to now, there are no specific products available for the treatment of sarcoptic mange in camelids and all the reports about effective treatments are involving a long period of time (2, 6, 8). Here, we report two clinical scenarios of sarcoptic mange: a case of an infestation with *Sarcoptes* mites in an alpaca with severe ear skin lesions from a zoo in Romania and its progress after successful treatment with a combination of amitraz and injectable eprinomectin and a case of infestation with *Sarcoptes* mites in two adult llamas from Italy and successful treatment with 2% subcutaneous moxidectin.

## Materials and Methods

### Case 1

A 5-year-old female alpaca (Bonnie) was brought to our attention by the animal care worker who noticed some dermatological problems. The alpaca was part of a three-animal group (two females and one male) from the public zoo in Galaţi, south-eastern Romania. The animals were housed together in an outside paddock with free access to an inside shelter. The animal was donated to the zoo, together with another alpaca by its former private owner who kept it as a pet. Both donated alpacas were kept in quarantine for 14 days before introducing them in the paddock with the other alpaca from the zoo. As part of the routine parasite control program, all alpacas were given an annual subcutaneous shot of ivermectin (1 mL/50 kg), with the last dose was given in September 2019. In August 2020, the zoo veterinarian performed a clinical examination of the herd and skin lesions on the ears accompanied by restlessness were observed in one of the animals. No other clinical signs were noted, and no other animals were clinically affected. The patient was restrained and a skin sample containing crusts, scabs and plucked hair was collected and sent to the Department of Parasitology and Parasitic Diseases at USAMV Cluj-Napoca. The skin sample underwent a direct examination under a stereo zoom microscope. In addition, artificial digestion of the crusts, using 10% KOH was performed. The collected crusts were mixed with 200 mL of 10% potassium hydroxide solution and heated until boiling. The digestion was interrupted after 3 min, when the skin particles were dissolved. The material was transferred to 50 mL corning tubes and centrifuged for 2 min at 1,000 rpm. The supernatant was discharged, and the sediment was poured into a Petri dish. Mites were cleared from the skin crusts and analyzed under an optical microscope (Olympus BX61). Part of the mites was mounted on slides with lactophenol and morphologically identified. Treatment with doramectin (Dectomax, Zoetis, US) 1 mL/50 kg was injected subcutaneously four times at 2-week intervals. The other two alpacas were also treated even though they did not present any lesions. Every 2 weeks, all the animals from the same group were visually inspected in order to follow the evolution of the disease. Since no improvement was noted, after 2 months, the alpaca with the clinical disease was transferred to the Department of Parasitology and Parasitic Diseases, USAMV Cluj-Napoca. A complete physical examination was performed, followed by hematology and blood biochemistry. Two cutaneous samples were collected using sterile swabs for detection of secondary fungal and bacterial associated infections using specific culture media. A multivitamins and minerals complex (Selevit, Pasteur Institute, Romania, 5 mL/animal) was orally administered daily for 2 weeks in order to help the recovery and acclimatization to the new environment. Local treatment with salicylic acid (BingoSpa, Poland) (100%) mixed with glycerol (BingoSpa, Poland) (99.5%) was applied for five consecutive days for removing the hyperkeratotic crusts. The ear pinas were washed weekly with chlorhexidine 3% shampoo (DouxoS3, Ceva Sante Animale, UK) for the whole period of the treatment, followed by local application of amitraz (0.5 mL diluted in 200 mL water, 12.5% Scobatox, Pasteur, Romania) using a clean sponge. Eprinomectin (Eprecis, Ceva Sante Animale, UK) 0.6 mL (1 mL/100 kg) was administered subcutaneously weekly for 8 weeks. Every week, the animal was removed from the shelter for a cleansing protocol and 5 g tetramethrin + 50 g transmix (Neostomosan, Ceva Sante Animale, UK) (1:200 dilution) was sprayed in the room. Skin scrapings were collected every week to evaluate the efficacy of the treatment, until negative for 2 months. After the complete recovery, the animal was sent back to the zoo.

The animal was legally transported from Galaţi to Cluj-Napoca, regarding the national legislation. Approval of the bioethical committee was not necessary since we did not do any experimental procedures on it. During its stay in Cluj-Napoca, the animal was housed accordingly.

### Case 2

The second episode refers to a couple of adult llamas, a 45 kg 4-year-old female (Pacha) and a 40 kg yearling male (Cuzco), owned by the managers of a recreational teaching farm in Caselette, north-western Italy. Like the fore mentioned alpaca, patients were part of a three-animal group including an adult female goat, housed in a paddock with free access to an indoor shelter. Both llamas were purchased from a nearby llama farm on April 3rd, 2019. According to owners, on arrival both animals appeared in excellent body condition and no skin lesions nor pruritus were observed. No quarantine nor any anti-parasitic treatment were applied before putting llamas in contact with the single adult goat already inhabiting the paddock. In mid-May, owners recorded itching in all animals and obvious alopecia and reddening on the distal hindlimbs of the male llama. Based on contagiousness and symptoms, a local large animal practitioner clinically suspected “mange” and applied to all animals a twofold subcutaneous treatment with 2% ivermectin (Ivomec®, Boehringer Ingelheim) at the approximate dose of 200 μg/kg b.w. at 11-days intervals. Since no clinical improvement was observed in llamas, a third dose of ivermectin treatment was administered after 8 days, with poor results. This prompted the request for a second opinion by the Parasitology unit of the Department of Veterinary Sciences, University of Turin. Patients were first visited on site on June 25th. Itching was noticeable in all animals in the paddock. Both, the llamas, and the goat were physically restrained, and, besides recording and documenting skin lesions, deep skin scrapings were obtained from multiple sites at the margins of affected skin. Collected samples were digested in a 10% KOH solution for 2 h at room temperature, and numerous *Sarcoptes* mites of all stages were microscopically observed in all scrapings from llamas though not from the female goat. A new therapeutic plan was suggested limited to clinically affected llamas, including an initial twofold subcutaneous administration of 2% moxidectin (Cydectin®, Virbac, Italy) at the approximate dose of 600 μg/kg b.w. at approximately 10-days apart, eventually followed by similar additional administrations in the event of persistent mite positive scrapings independently of clinical improvement. It was established that the treatment plan would have not been suspended before a second serial negative skin scraping would have been obtained from both llamas.

Ethics approval was deemed unnecessary.

## Results

### Case 1

**Figure 1:**
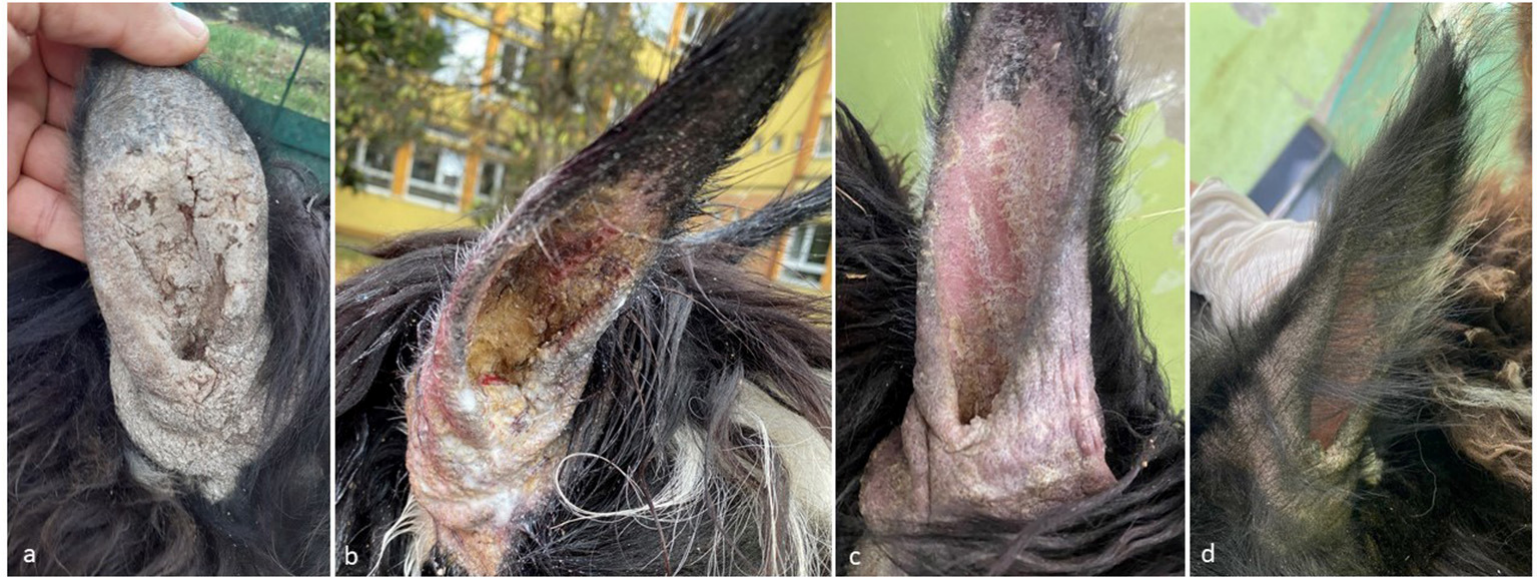
Alpaca (case no. 1), ear pinnae. Evolution of skin lesions from severe crusting dermatitis, hyperkeratosis, focal ulceration, alopecia, and scaling **(a)** to moderate to severe crusting, hyperkeratosis, scaling, erythema and alopecia **(b)** to mild to moderate alopecia, erythema and scaling **(c)** to mild alopecia and erythema **(d)**.

**Figure 2:**
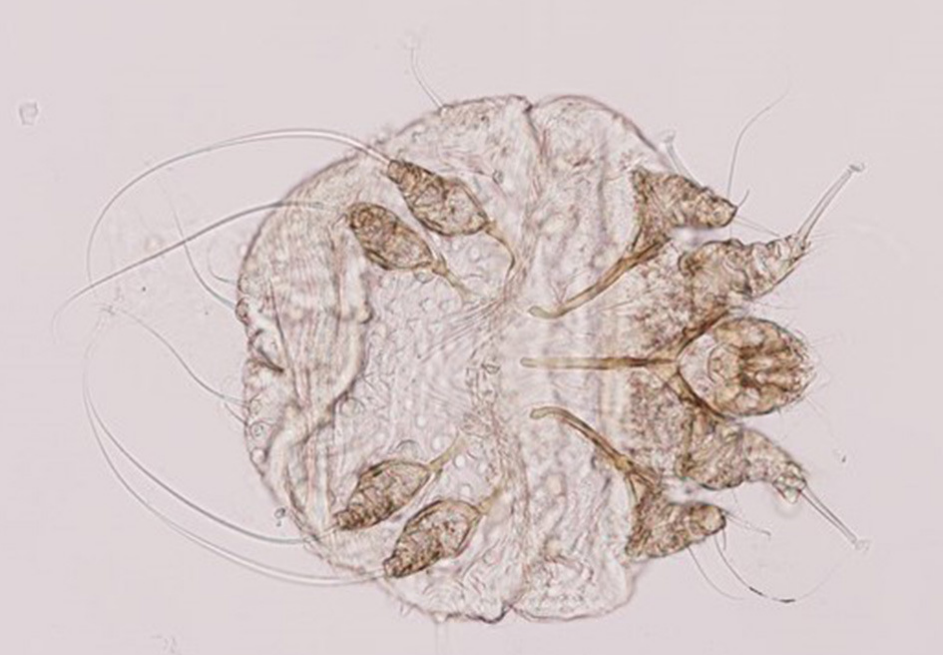
Morphological features of *Sarcoptes scabiei* mites.

Clinical inspection revealed extreme agitation due to pruritus and the presence of scaly, crusty lesions on the external ears accompanied by local alopecia, hyperkeratosis, and thickened skin (Figure 1a). The direct examination of the collected skin sample showed no evidence of a parasitic infestation, but mites were detected after being freed from the galleries by performing the digestion of the skin crusts. The mites were morphologically identified as *Sarcoptes scabiei* (Figure 2). Different stages of *Sarcoptes* were detected: eggs, larvae, adult females, and males. No other mites were detected. Blood biochemistry and hematology showed no abnormal values. Secondary infections with bacteria or fungi were not detected.

Two weeks after the first treatment, the lesions failed to improve. However, crusts were almost completely removed after 10 days of topical application of 3% chlorhexidine shampoo and the salicylic acid. After 1 month from the combined antiparasitic treatment in the new location, alive and dead mites were still present in the skin scrapings. After another two weeks, all the detected mites were dead and by the end of the treatment (8 weeks), no mites were detected. The monthly evolution of the skin lesions is presented in Figure 1. None of the people involved in handling the alpaca, nor the zookeepers manifested any pruritus or other dermatological conditions. The animal was followed by phone, by questioning the zoo owner. After 5 months, no relapse was noticed.

### Case 2

**Figure 3:**
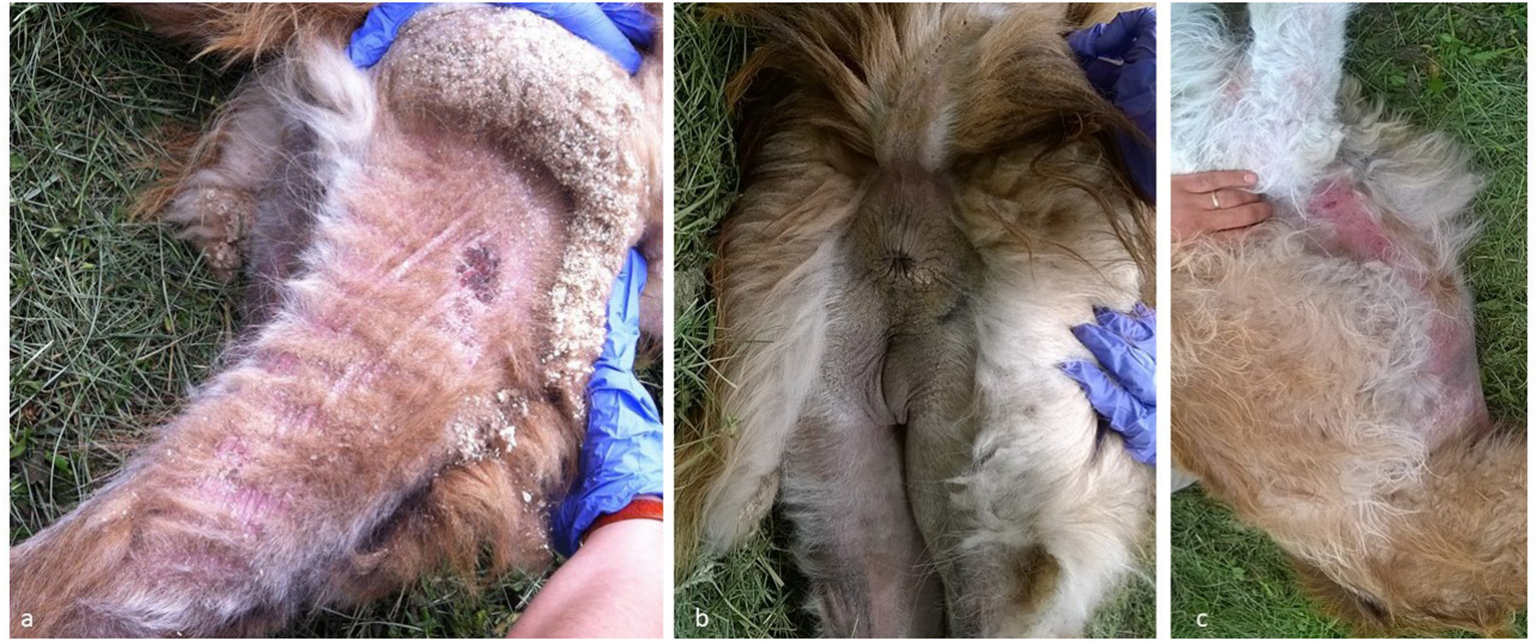
Llamas (case no. 2), skin lesions. **(a)** Hind limb (after clipping): alopecia, moderate to severe erythema, severe scaling and focal crusts; **(b)** Hind limbs, perianal, peri vulvar region and ventral aspect of the tail: alopecia, mild erythema and scaling and mild, focal lichenification; **(c)** Ventral abdomen and axillae: extensive alopecia, moderate to severe erythema and focal hyperpigmentation.

At first consultation (Day 0), severe itching was obvious on both llamas though skin lesions were far more extensive in Cuzco, including: severe, diffuse scaling, particularly noticeable after hair clipping; skin reddening on the belly, axillae and, to a lower extent, on forelegs and hind limbs; hair thinning to alopecia on the belly, axillae, forelegs, distal fore and hind limbs, posterior and internal aspects of the proximal hind limbs; skin wrinkling on the last mentioned areas on the hind limbs; soft and relatively thin (≤ 2 mm) exudative crusts on medial cantus of the eye, the ventral aspect of the tail, the perianal zone and the perineum (Figure 3). Skin lesions were more localized in Pacha, in which reddening and hypotrichosis to alopecia, on the axillae, forelimbs, external pinnae and internal aspect of the thighs, and thin, soft (≤1 mm) crusts in the perianal zone were recorded. Trivial dermatological signs were observed in the cohabiting goat, including mild pruritus, a dull coat and small scattered alopecic areas on the neck and shoulders, possibly because of scratching. At second consultation (Day 10 post-treatment–PT), after the treatment, signs had disappeared in the goat though not in the llamas, in which severe itching was persistently noticed by owners in the time interval from the first moxidectin administration. On handling, the only clinical improvement was the attenuation of skin reddening on Cuzco's belly and axillae and the initial drying of the localized crusty lesions in both llamas. Skin scrapings remained positive in both patients, whereas the recovered goat was not sampled. Mites of all stages were observed in Cuzco, though only eggshells, larvae and a few nymphs were present in Pacha. At third consultation (Day 19 PT), after the second dose of moxidectin, mild itching was observed only in Cuzco. In this patient, a general attenuation of scaling and skin reddening was present, together with the disappearance of crusty lesions in the perianal and perineal zone and their further drying in the periocular zone. In Pacha, clinical improvement was evident, including the disappearance of skin reddening, clearance of perianal crusty lesions and the observation of initial hair regrowth on the previously alopecic area. At skin scraping, no mites were detected in Pacha whereas larvae, eggshells and a few larvated eggs and nymphs were still observed in Cuzco. A third dose of moxidectin was administered to both llamas and at fourth consultation (Day 30 PT) no itching was recorded on any animals nor any skin alterations were detectable on Pacha and the goat. In Cuzco, crusts on the periocular zone had cleared and the only persisting alterations were attenuated skin wrinkling on the posterior aspect of the proximal hind limbs and the incomplete hair regrowth in previously alopecic zones. No mites were present in the skin scrapings of both llamas. A fourth dose of moxidectin was administered to them followed by the fifth and final consultation (Day 40 PT), with no clinical signs detected on any of the animals. A lively hair regrowth of previously alopecic areas was present in Cuzco. Skin scrapings blindly obtained from previously affected areas of both llamas yielded negative results. According to protocol, no further treatments were applied. Periodic phone contacts with the llama owners in the 12 months that followed the closure of the case witnessed no relapse and a healthy state in all animals.

## Discussion

Sarcoptic mange in llamas and alpacas represents a severe, sometimes lethal, dermatological condition (9–11). In various wildlife, *S. scabiei* is responsible for high morbidity and mortality (12). Treatment of scabies in camelids is challenging, with no specific labeled products available in Europe. Several molecules were used in the treatment of sarcoptic mange in alpacas, with low to moderate efficacy (2, 10). In one study (10), three alpacas had to be euthanized as one of the same groups died and the others did not respond to treatment with three doses of doramectin, nor with three doses of ivermectin, amitraz or diazinon (10). Repeated treatment with subcutaneous 0.2 mg/kg ivermectin was reported as effective in treating sarcoptic mange in alpacas, but after long-term administration of at least eight doses (11). However, ivermectin was not suitable in none of our present cases for treating sarcoptic mange. Topical treatment with amitraz was successfully used in three *S. scabiei*-infected alpacas from UK with travel history, after the diseases failed to respond to topical eprinomectin administered in several doses (13). In Belgium, a severe case of sarcoptic mange in an alpaca was finally treated after 3 years of struggle, with 10% moxidectin (1 mg/kg) long-acting subcutaneous injection (14). A similar situation was presented in our case 2, from Italy, where successful treatment was achieved by using long-term high dose moxidectin. However, in other studies, topical treatment with doramectin, eprinomectin or moxidectin had limited efficacy in treating sarcoptic mange in alpacas (7, 15–17). This may be due to hyperkeratosis, a typical lesion developed in alpaca affected by sarcoptic mange which can influence the penetration and absorption of topical molecules (6). Another cause suggested for the poor efficacy of topical acaricides is the lack of lanolin (the woold grease), which can limit their efficacy (6, 18). Considering these, in the first case study we used a combined treatment with topical amitraz and subcutaneous eprinomectin, only after the thick crusts were removed with keratolytics in order to facilitate the absorption of the topical acaricide. However, we used this protocol on only one patient, and it could be very time consuming and costly in the case of bigger herds. In another study, the successful treatment of a mixed infestation with sarcoptic and chorioptic mange mites in one alpaca was achieved using a combination of topical amitraz and 0.5 mg/kg subcutaneous ivermectin for nine consecutive weeks (6). Combined treatments seem to be more efficient, especially when associated with local shampooing with chlorhexidine, vitamins, and environmental treatment (2, 6). Pour-on eprinomectin (0.5 mg/kg) was used in the treatment of chorioptic mange in alpacas with (15) or without (16) success. Only in one of all these studies, the animals were followed for a longer period (that is about one year) in order to be sure that the disease did not recur (13). Unfortunately, all the effective treatments reported until now are time consuming and take a long period of time. The elaboration of specific products, with a faster period of treatment would greatly improve the present situation. In both cases reported in this study, the source of the infection is unknown. In the second case, it is unlikely that infection originated from the single domestic goat with which llamas were put into cohabitation, since authors' clinical experience and a previous regional serological survey suggest that is extremely rare that exposure to *Sarcoptes* infection occurs in domestic goats in Northern Italy (19). Similarly, as in other models (20, 21), molecular epidemiological studies are advisable to clarify if sarcoptic scabies episodes in South American camelids raised in Europe are traceable back to spill over from other domestic reservoirs or derive from the circulation of imported *Sarcoptes* strains adapted to these specific hosts.

To the best of our knowledge, we present in this paper two new effective therapeutic protocols for the treatment of sarcoptic mange in European camelids.

## Conclusion

We present two clinical scenarios of sarcoptic mange, one in an alpaca from a zoo in Romania, and one in two llamas from a farm in Italy, successfully treated with a combination of 3% chlorhexidine, keratolytics, followed by ectocides (topical amitraz and subcutaneous eprinomectin) and 2% subcutaneous moxidectin, respectively.

## Data Availability Statement

The original contributions presented in the study are included in the article/supplementary material, further inquiries can be directed to the corresponding authors.

## Ethics Statement

Ethical review and approval was not required for the animal study because It was deemed unnecessary. Written informed consent for participation was not obtained from the owners because Verbal informed consent was enough as the owner (zoo director) came with the animal to our clinic for treatment.

## Author Contributions

GD was responsible of diagnosis and treatment and wrote the manuscript. BM offered support in the diagnosis of the animals and help with the writing of the manuscript. AMB contributed to the description of lesions and offered specialized advices in choosing the treatment. LRa and LRo were responsible for the diagnosis treatment and follow-up of the second case presented. ADM supported this study and revised the manuscript. All authors contributed to the article and approved the submitted version.

## Funding

The main author, GD, was financially supported by Altius SRL Romania as part of her EVPC residency. Eprinomectin and the chlorhexidine shampoo was kindly provided by Ceva Sante Animale Romania. The paper was published under the frame of European Social Found, Human Capital Operational Programme 2014–2020, project no. POCU/380/6/13/125171.

## Conflict of Interest

The authors declare that the research was conducted in the absence of any commercial or financial relationships that could be construed as a potential conflict of interest.

## Publisher's Note

All claims expressed in this article are solely those of the authors and do not necessarily represent those of their affiliated organizations, or those of the publisher, the editors and the reviewers. Any product that may be evaluated in this article, or claim that may be made by its manufacturer, is not guaranteed or endorsed by the publisher.
